# Electroreduction of CO_2_ in a Non-aqueous Electrolyte—The Generic Role of Acetonitrile

**DOI:** 10.1021/acscatal.3c00236

**Published:** 2023-04-13

**Authors:** Thomas Mairegger, Haobo Li, Christoph Grießer, Daniel Winkler, Jakob Filser, Nicolas G. Hörmann, Karsten Reuter, Julia Kunze-Liebhäuser

**Affiliations:** † Department of Physical Chemistry, University of Innsbruck, Innrain 52c, Innsbruck 6020, Austria; ‡ School of Chemical Engineering, University of Adelaide, Adelaide 5005, Australia; § Theory Department, Fritz-Haber-Institut der Max-Planck-Gesellschaft, Faradayweg 4-6, Berlin 14195, Germany

**Keywords:** electrocatalysis, electrochemical CO_2_ reduction, acetonitrile, infrared spectroscopy, molybdenum carbide, carbon monoxide

## Abstract

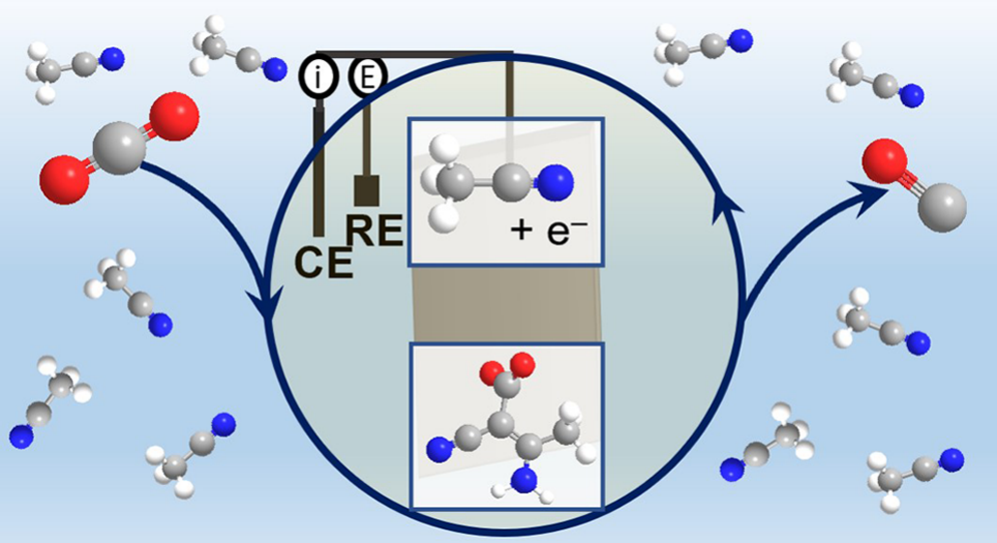

Transition metal carbides, especially Mo_2_C, are praised to be efficient electrocatalysts to reduce CO_2_ to valuable hydrocarbons. However, on Mo_2_C in an aqueous electrolyte, exclusively the competing hydrogen evolution reaction takes place, and this discrepancy to theory was traced back to the formation of a thin oxide layer at the electrode surface. Here, we study the CO_2_ reduction activity at Mo_2_C in a non-aqueous electrolyte to avoid such passivation and to determine products and the CO_2_ reduction reaction pathway. We find a tendency of CO_2_ to reduce to carbon monoxide. This process is inevitably coupled with the decomposition of acetonitrile to a 3-aminocrotonitrile anion. Furthermore, a unique behavior of the non-aqueous acetonitrile electrolyte is found, where the electrolyte, instead of the electrocatalyst, governs the catalytic selectivity of the CO_2_ reduction. This is evidenced by in situ electrochemical infrared spectroscopy on different electrocatalysts as well as by density functional theory calculations.

## 1 Introduction

The CO_2_ concentration in the atmosphere has reached a new record high of around 420 ppm^1^ and is caused by humanity’s industrial metabolism, which is a perturbation to Earth’s natural carbon cycle.^2^ To limit global warming below 2 °C, compared to pre-industrial levels, an interplay between different technologies such as decarbonization, carbon sequestration, and carbon recycling is necessary.

Value-added products in the electrochemical CO_2_ reduction reaction (CO_2_RR), such as methanol, methane, or ethylene, involve more than two electron-transfer steps, whereby each step increases the complexity of the reaction.^3−5^ Among metal electrocatalysts, only Cu or Cu-based materials were found to form these products with reasonable Faradaic efficiencies, while others form formic acid/formate, carbon monoxide, or hydrogen as the major product.^3−5^ However, low product selectivity and high overpotentials govern the reaction on Cu catalysts, which necessitates further research for better-suited electrocatalysts.

Since the number of possible electrocatalysts is vast, theoretical calculations are vital to narrow the search space and propose materials with high activity and selectivity toward the desired CO_2_RR products. In such theoretical studies, single-crystal transition metal carbide surfaces were proposed to be highly active due to their more oxophilic and carbophobic nature compared to their parent metals.^6^ Their chemical nature should enable them to break the scaling relations of key reaction intermediates, i.e., CO and CHO, that dominate the essential CO_2_RR steps.^7^ A detailed active site computational screening study has focused on the investigation of the various active sites of Mo_2_C and confirmed its high suitability for CO_2_RR.^8^

Despite the highly praised activity of Mo_2_C, experimental studies in an aqueous electrolyte never confirmed the formation of the expected products and clearly showed that the competing hydrogen evolution reaction (HER) is favored over the whole potential range of interest.^9^ The immediate surface oxidation of the electrocatalyst, which occurs upon exposure to air and even by immersion into an aqueous electrolyte, was subsequently found responsible for this preferred formation of H_2_.^9^

To avoid the passivation of the surface and circumvent the concomitant high HER activity, we here change the reaction conditions to a non-aqueous electrolyte and simultaneously circumvent electrode contact to an ambient atmosphere. The chosen acetonitrile-based solution allows working over a wider potential window due to its higher stability and shows a higher CO_2_ solubility than any other aqueous electrolyte.^10^ Under these reaction conditions, the non- or barely oxidized Mo_2_C is able to reduce CO_2_, which is confirmed via in situ infrared spectroscopy (IR). However, no higher reduced products are found, which is in clear contrast to the theoretical predictions. Intriguingly, we furthermore find that all sorts of investigated electrode materials interfaced with the acetonitrile-based electrolyte yield identical product distributions. This implies that the impact of the acetonitrile electrolyte is much higher than previously thought, not to say decisive for the reaction route. The electrocatalyst seems instead to only act as an electron donor, while the selectivity is independent of the catalyst material.

## 2 Experimental Section

### 2.1 Synthesis of Mo_2_C

The polycrystalline electrocatalyst was synthesized according to previous publications.^9,11^ Here, Mo (99.95%, Advent Ltd.) carburization was performed in a home-built, vacuum-assisted quartz furnace, which allows the transfer of electrodes into the glovebox under a H_2_ atmosphere after the synthesis.

### 2.2 Electrochemistry

All electrochemistry measurements were performed in an Ar-filled glovebox at room temperature in an acetonitrile electrolyte containing 0.1 M tetrabutylammonium hexafluorophosphate (TBAPF_6_) utilizing an Autolab (Metrohm) potentiostat. The potentials are given versus the ferrocene/ferrocenium couple,^12^ abbreviated as *V*_Fc/Fc+_. In Figure S1, the half-wave potential in acetonitrile with 0.1 M TBAPF_6_ was determined. The details are outlined in Note 1.

### 2.3 Electrochemical Infrared Spectroscopy

All electrochemical infrared reflection absorption spectroscopy (EC-IRRAS) measurements were carried out in a home-built three-electrode spectroelectrochemical cell at room temperature with a VERTEX 70v spectrometer (Bruker). The IR cell was assembled in the glovebox and transported to the IR spectrometer under air exclusion. Details are given in Note 1 and in ref (13).

### 2.4 DFT Calculations

DFT calculations were performed with the plane-wave basis, pseudopotential package QuantumESPRESSO (QE),^14,15^ using the van der Waals-corrected BEEF-vdW exchange–correlation functional.^16^ Surface structures were modeled at fully optimized symmetric slabs separated by a vacuum region of 20 Å. Using a plane-wave cut-off of 800 eV, all structures were fully relaxed until residual forces fell below 0.03 eV/Å. Test calculations with higher cut-offs and *k*-point grids indicate the obtained surface free energies to be converged within 10 and 1 meV/Å^2^, respectively.^9^ Adsorption structures with small size adsorbates (CO, CO_2_, COH, CHO, and COOH) were calculated with a (1 × 1) unit cell and a (4 × 4 × 1) *k*-grid, while large size adsorbates (CO_3_, C_2_O_2_, C_2_O_4_, HCO_3_, and HCO_2_) were calculated with a (2 × 2) unit cell and a (2 × 2 × 1) *k*-grid. The small gas-phase molecules (H_2_, H_2_O, and CO_2_) were calculated for electronic energies and vibrational frequencies separately in each supercell with a side length of 10 Å, while the larger species in the solvent (3-aminocrotonitrile anion, 3-aminocrotonitrile, carboxylated acetonitrile, and carboxylated 3-aminocrotonitrile anion) were calculated for vibrational frequencies in each supercell with a side length of 20 Å.

## 3 Results and Discussion

### 3.1 CO_2_ Electroreduction Performance at Mo_2_C in Non-aqueous Media

**Figure 1:**
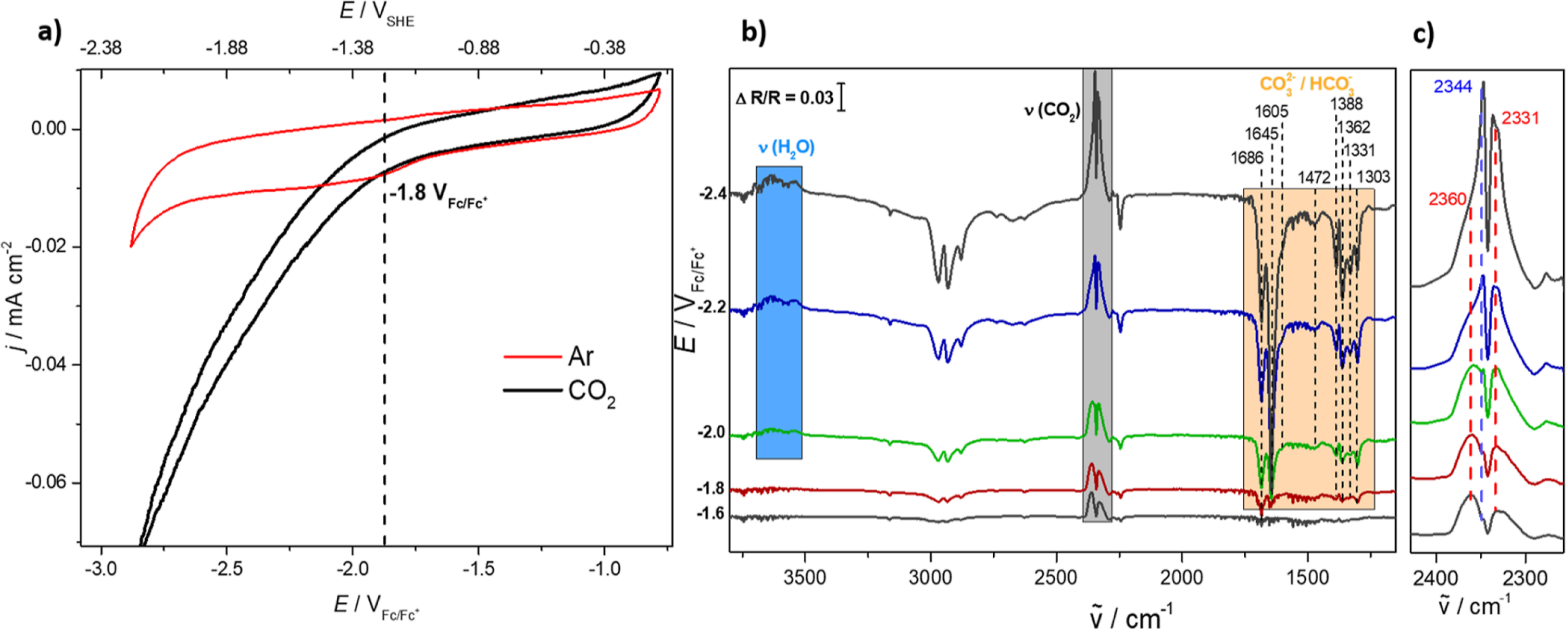
(a) CVs of Mo_2_C recorded in Ar-purged (red) and CO_2_-saturated (black) acetonitrile with 0.1 M TBAPF_6_. Scan rate: 50 mV/s. (b) EC-IRRA spectra for the CO_2_ reduction at Mo_2_C in CO_2_-saturated acetonitrile with 0.1 M TBAPF_6_. The spectra were recorded in cathodic direction (step potential: bottom to top). The spectra show the consumption of CO_2_ (gray box) and residual water (blue box) in the electrolyte and the formation of carbonate/bicarbonate species (orange box) due to water reduction. The reference potential was at −1.0 V_Fc/Fc+_ (see the Supporting Information for details). All the non-highlighted bands are associated with TBAPF_6_ or acetonitrile since they also appear in the reference spectrum (Figure S2). (c) Enlarged view of the CO_2_ region to distinguish the dissolved CO_2_ band (2344 cm^–1^) from the R- and P-branches of gaseous CO_2_ (2360 and 2331 cm^–1^).

Electrochemistry in combination with EC-IRRAS provides a general picture of the CO_2_RR behavior of the carbide material in non-aqueous media. The cathodic scan of the cyclovoltammogram (CV) of Mo_2_C recorded in CO_2_-saturated acetonitrile with 0.1 M tetrabutylammonium hexafluorophosphate (TBAPF_6_) (Figure 1a, black) shows an earlier increase of the negative current density than the respective scan in an Ar-purged electrolyte (Figure 1a, red). This sparks the conjecture that the increasing current is due to the formation of products from CO_2_RR. To determine the nature of these products, EC-IRRAS studies were conducted that allow us to monitor the formation (downward facing bands) and consumption (upward facing bands) of species in situ at the respective applied potential. The details of the EC-IRRAS experiments are outlined in Note 1.

Spectra were recorded in both degassed (Figure S2) and CO_2_-saturated (Figure 1b) acetonitrile with 0.1 M TBAPF_6_ to identify the bands associated with the electrolyte and with the CO_2_RR products. Additional features clearly appear in the CO_2_-saturated solution (Figure 1b, highlighted in color) compared to the reference (Figure S2), while the bands for TBA^+^ disappear. The additional features occur at potentials < −1.80 V_Fc/Fc+_ (corresponding to −1.18 V_SHE_),^17^ which is in perfect alignment with the CVs (Figure 1a, black), in which a nominal onset of −1.80 V_Fc/Fc+_ for the CO_2_ reduction was determined. The disappearance of the TBA^+^ bands has been observed in every spectrum and has also been reported in the literature.^18−20^ Currently, we have no explicit explanation for this behavior.

The assignment of these product bands for CO_2_RR (Figure 1b; orange box) is, however, not straightforward and is also still debated in the literature.^18−20^ In agreement with Figueiredo et al.,^20^ we assign the bands between 1700 and 1300 cm^–1^ to carbonate/bicarbonate formation due to visible water consumption (Figure 1b; blue box) of the residual water in the electrolyte (1) and the CO_2_ depletion (Figure 1b, gray box). The band of dissolved CO_2_ (2343 cm^–1^) starts to form at −1.8 V_Fc/Fc+_ in the center of the two R- and P-branches of gaseous CO_2_ (Figure 1c, 2360 and 2331 cm^–1^) that result from insufficient purging of the spectrometer.
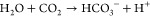
1
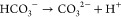
2
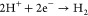
3
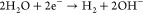
4
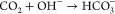
5

Despite the significant depletion of CO_2_ (Figure 1b, gray box) and formation of carbonates/bicarbonates at potentials between −1.8 and −2.4 V_Fc/Fc+_, this is not related to the electrochemical reduction of CO_2_ to CO and CO_3_^2–^, as reported for organic electrolytes in the literature;^21−23^ as the key feature, a band related to the formation of CO is not observed. In organic electrolytes, either proton (eq 3) or water reduction (eqs 4 and 5) can take place and is visible as HER. In the present work, in the potential range investigated, the HER is only detectable with CO_2_ in the electrolyte (compare Figures 1b to S2) due to the proton reduction initiated by the chemical reaction of CO_2_ with water to H^+^ and HCO_3_^–^/CO_3_^2–^ (eqs 1 and 2). This reaction shifts toward the products due to the electroreduction of H^+^ to hydrogen (eq 3).

### 3.2 Binding of Intermediates

**Figure 2:**
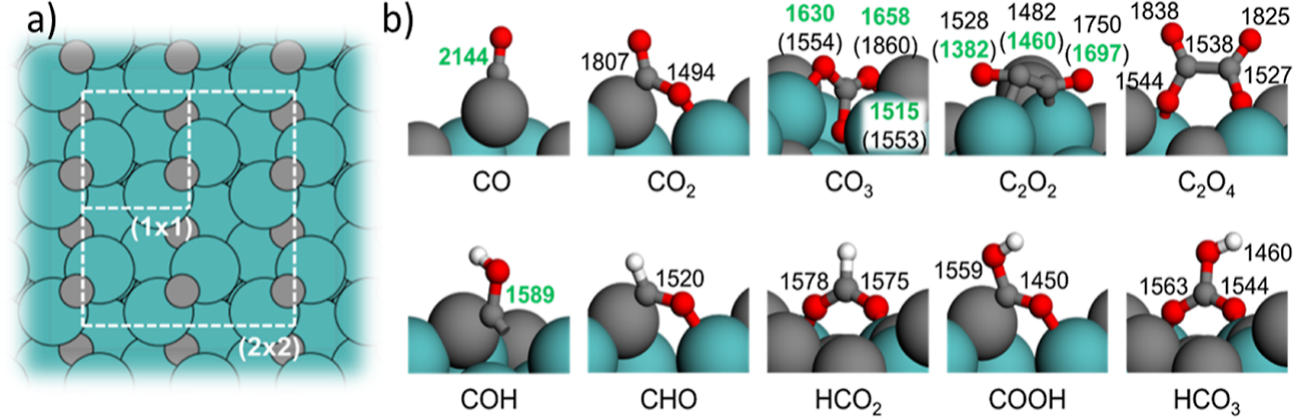
(a) Top view of the C-rich Mo_2_C (110) surface, showing the employed (1 × 1) and (2 × 2) surface unit cells as white rectangles for the calculation of the small and large size adsorbates, respectively. (b) Most stable adsorbate configurations on C-rich Mo_2_C (110). Offset-corrected DFT vibrational frequencies in acetonitrile are given at each C–O or C–C bond (unit: cm^–1^). Due to the small energy differences, the frequencies of the second most stable adsorption configuration are also provided in parentheses for C_2_O_2_ and CO_3_. Large green spheres: Mo; gray spheres: C; red spheres: O; and white spheres: H.

A strong indication for the fact that all the formed species are present inside the electrolyte solution and not adsorbed at the electrode surface is that none of the bands show a frequency shift with the applied potential (Figure 1b). To further strengthen this assertion, IR investigations with p- and s-polarized light were conducted. They revealed no significant differences between the spectra (Figure S3), which emphasizes that all the products are in fact species in solution. This notion is further supported by independently performed extensive calculations of vibrational modes of widely conceivable reaction intermediates and products at the prevailing Mo_2_C(110) facet.^9^ None of these vibrations, summarized in Figure 2b, together with the corresponding intermediates (an overview of all calculated species is given in Note 2, Tables S1–S3, and Figures S7 and S8) match the spectral response obtained during the experiment.

The final proof to exclude a possible chemical binding of the intermediates to the electrode is that the spectra recorded in Figure 1b perfectly agree with the literature IR data^18−20^ for different electrocatalysts studied in the same acetonitrile electrolyte. As it is unlikely that different catalysts with their differing binding strengths and concomitantly changed reaction pathways and adsorbate configurations all lead to the same specific adsorption bands,^24^ we conclude that these bands instead arise from species in solution.

### 3.3 CO_2_ Electroreduction and Acetonitrile Decomposition

At potentials ≤ −2.4 V_Fc/Fc+_, acetonitrile starts to decompose when no CO_2_ is present (Figure S4). In this process, the acetonitrile anion, which is formed due to the deprotonation of acetonitrile by a hydride ion from the metal lattice, nucleophilically attacks a second acetonitrile molecule to form the 3-aminocrotonitrile anion.^25^ The most prominent features that indicate the presence of the 3-aminocrotonitrile anion are the bands at 2118 and 1517 cm^–1^, as well as weaker bands below 1350 cm^–1^ (Figure S4).^25^ Stepping back to more anodic potentials (−1.0 V_Fc/Fc+_) results in protonation of the anion,^25^ which is confirmed by a blue shift of the band from 2118 to 2180 cm^–1^ and by the formation of new bands at around 3400 and 1600 cm^–1^ (Figure S5).^25^ In the case of CO_2_-saturated acetonitrile-based solution, however, electrolyte decomposition is not the only reaction occurring.

**Figure 3:**
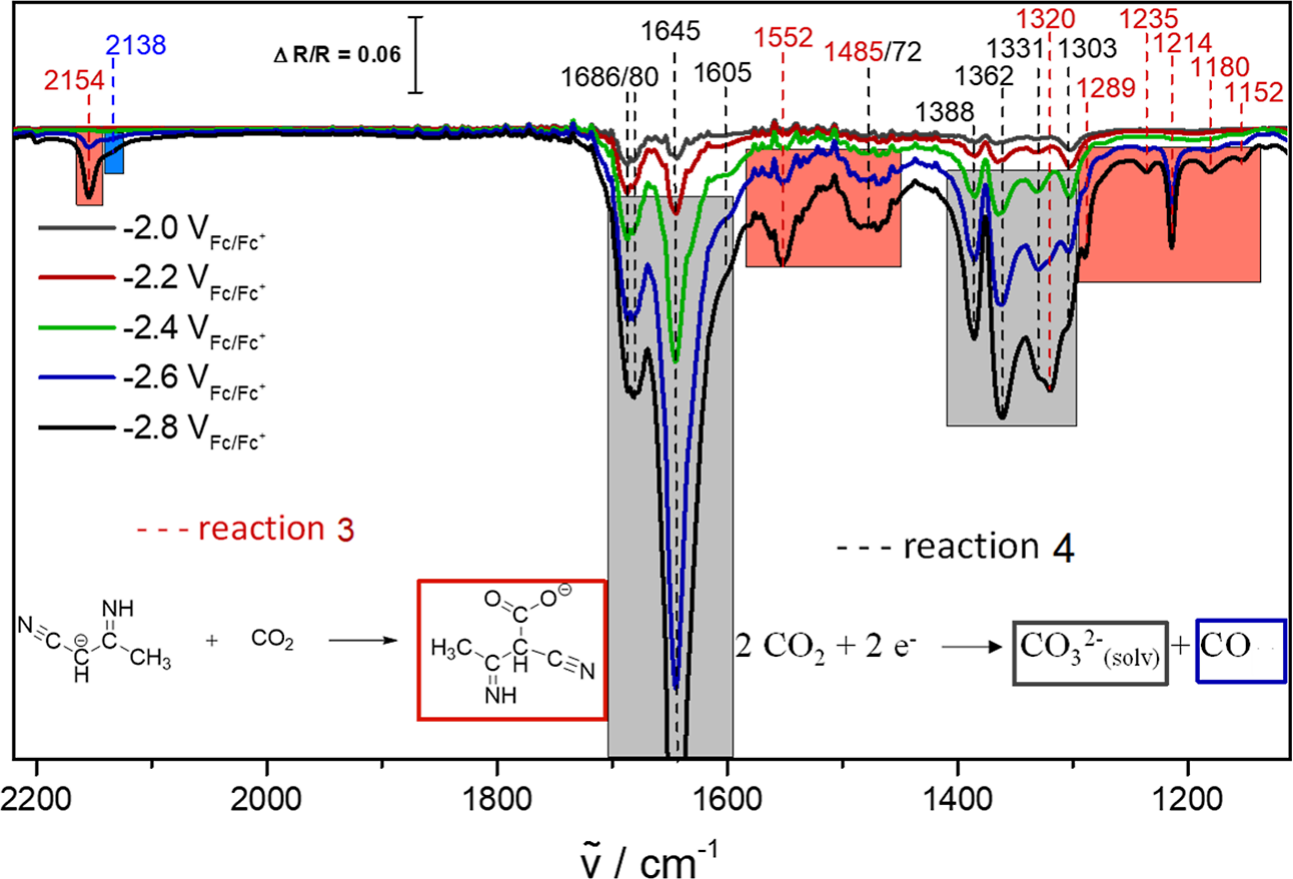
EC-IRRA spectra at Mo_2_C in CO_2_-saturated acetonitrile with 0.1 M TBAPF_6_. The reaction of the 3-aminocrotonitrile anion with CO_2_ (reaction 3, red boxes) takes place simultaneously with the disproportionation reaction of CO_2_ to dissolved CO and carbonate (reaction 4, blue and black boxes) at potentials below −2.4 V_Fc/Fc+_. The reference spectrum was at −1.0 V_Fc/Fc+_.

Indeed, in a CO_2_-saturated electrolyte (Figure 3), more bands appear at wavenumbers below 1300 cm^–1^. Additionally, no distinct bands at 2118 and 2180 cm^–1^, related to the 3-aminocrotonitrile anion (Figure S4) and its protonated form (Figure S5), are observed. This suggests that instead of acetonitrile decomposition, CO_2_ reduction occurs, which comes along with the formation of the respective reduction products. The detected IR bands suggest the formation of the 3-aminocrotonitrile anion and its instantaneous reaction with CO_2_ to form a carboxylated species (see reaction 3 in Figure 3). The carboxy group only occurs when CO_2_ is present and leads to a blue shift from 2118 cm^–1^ that is detected in an Ar-purged electrolyte (Figure S4) to 2154 cm^–1^ and to the formation of additional bands below 1600 cm^–1^ (Figure 3, red boxes).

Intriguingly, the bands assigned to carbonate and bicarbonate (Figure 3, gray boxes) are increasing more rapidly at potentials below −2.4 V_Fc/Fc+_, which cannot result from reaction 3. Furthermore, a new band at 2138 cm^–1^ (Figure 3, blue box) arises that can be assigned to the formation of dissolved CO.^20^ To validate this assignment, IR studies of the bare electrolyte purged with CO were performed in transmission, revealing a distinct band at 2138 cm^–1^ (Figure S6), which clearly proves the formation of CO at potentials below −2.4 V_Fc/Fc+_. The electrochemical reduction of CO_2_ to dissolved CO and solvated CO_3_^2–^ species is in perfect agreement with the distinctive increase of the carbonate bands and with the occurrence of a signal at 2138 cm^–1^. Hence, all features in Figure 3 can be related to reactions 3 and 4 (see the insets in Figure 3).

**Figure 4:**
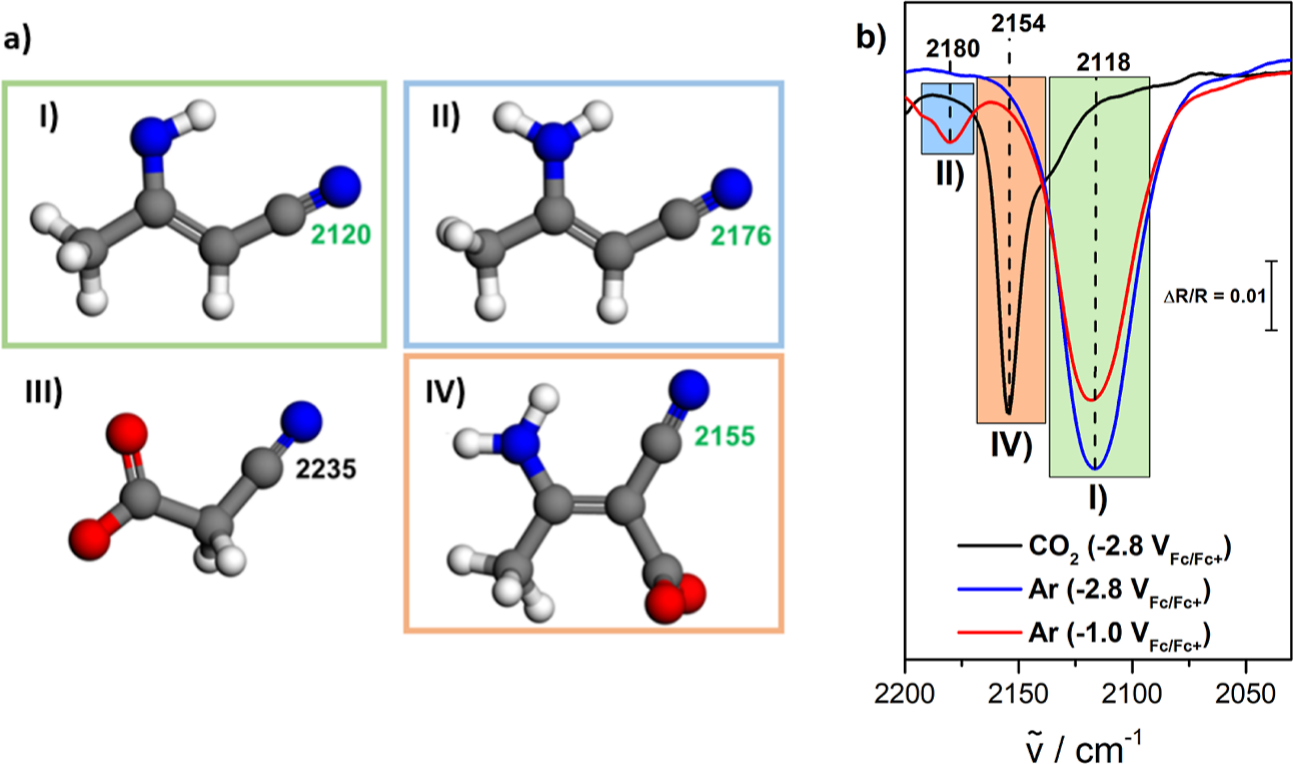
(a) Illustration and theoretically calculated wavenumbers of the (I) 3-aminocrotonitrile anion, (II) 3-aminocrotonitrile, (III) carboxylated acetonitrile, and (IV) carboxylated 3-aminocrotonitrile anion. Offset-corrected DFT vibrational frequencies (Table S4) are given for each species (unit: cm^–1^). Blue atoms: N; gray atoms: C; red atoms: O; and white atoms: H. (b) Enlarged view of the EC-IRRA spectra (extracted from Figures 3 and S5) in the wavenumber region between 2200 and 2000 cm^–1^. The theoretically calculated and experimentally measured wavenumbers align and prove the proposed reaction 3 (see Figure 3).

To confirm the proposed reaction of the anion with CO_2_, the relative shift of the wavenumbers among the 3-aminocrotonitrile anion, the 3-aminocrotonitrile, and the 3-aminocrotonitrile anion reacted with CO_2_ was calculated (Figure 4a). The calculated relative shifts are in exact alignment with the experimentally measured wavenumber shifts (Figure 4b), which validates the discussed reaction 3 (see Figure 3), where the 3-aminocrotonitrile anion (2120 cm^–1^) reacts with CO_2_.

The relative wavenumber of the carboxylated acetonitrile anion [Figure 4a(III)] was also calculated since it is an intermediate in the formation of the 3-aminocrotonitrile anion (see Figure S4). This wavenumber, however, is not experimentally measured, which suggests that this intermediate is too short-lived to be present in sufficient concentrations.

Mo_2_C is therefore clearly identified to be able to reduce CO_2_ to dissolved CO, while, simultaneously, acetonitrile decomposes. The decomposition product reacts further with CO_2_ and forms carboxylated 3-aminocrotonitrile (Figure 3, reaction 3).

### 3.4 Acetonitrile-Based Electrolyte Governs the Electrocatalytic Selectivity

The above proposed reaction pathway implies that the contribution of acetonitrile in the CO_2_ electroreduction is central. Even more intriguingly, in-depth literature research shows that all IR experiments in the CO_2_-saturated acetonitrile-based electrolyte yield the same spectral response, independent of the employed electrocatalyst (Cu, Pt, Au, Ag, Pb, or Pd).^18−20^ This suggests that the influence of the nature of the electrocatalyst is minimal compared to the influence of acetonitrile on the CO_2_RR selectivity.

**Figure 5:**
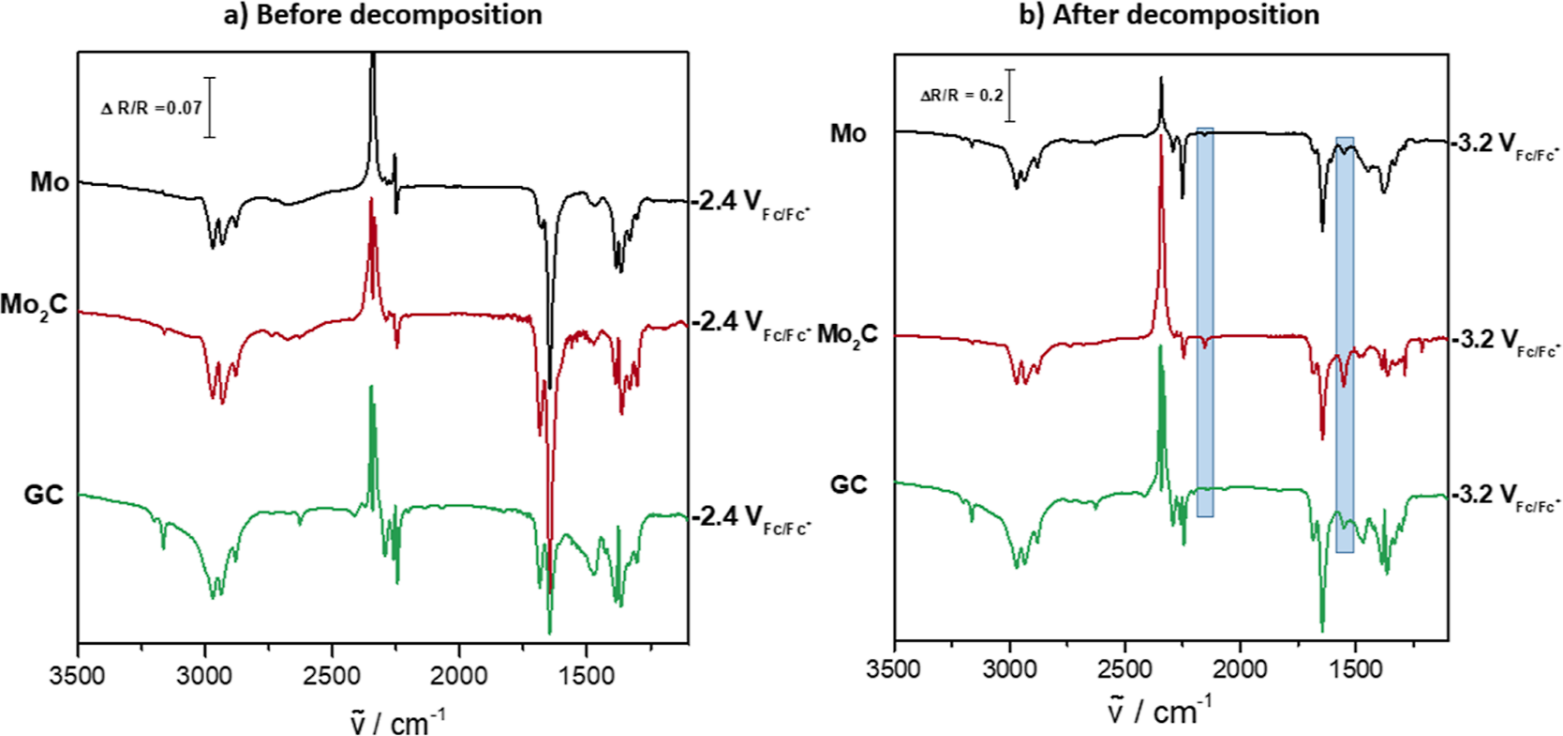
EC-IRRA spectra (a) before and (b) after the decomposition of acetonitrile for the CO_2_ reduction in the same electrolyte (acetonitrile + 0.1 M TBAPF_6_) at different electrodes. Mo (top, black), Mo_2_C (middle, red), and GC (bottom, green) were chosen as electrocatalysts. The comparison of all spectra, especially the identical band formation, emphasizes the central role of acetonitrile in the CO_2_ reduction selectivity. The potential for all reference spectra was −1.0 V_Fc/Fc+_.

To validate this assumption, IR investigations with different electrocatalysts were performed. The parent metal Mo was chosen to unravel possible differences to Mo_2_C with its theoretically praised high suitability for the CO_2_ reduction to higher reduced products. Additionally, a glassy carbon (GC) electrode was used as a rather inert reference material. All electrode materials show the exact same product distribution in the EC-IRRA spectra (Figure 5), which implies that the reaction is independent of the electrocatalyst material. It must therefore proceed in the same manner at the different electrode surfaces, which is supported by the fact that no specific adsorption occurs during the reaction. This is emphasized in Section 3.2, where the experimentally obtained wavenumbers are given that do not change with changing potential and that fit very well to the theoretically calculated wavenumbers of intermediates that are not adsorbed at the surface but existent in the solution. The intensities of the bands slightly differ between each catalyst, which is due to the slightly different reflectivities of the materials’ surfaces and small variations in the thin-layer thickness. This proves true not only for the electrocatalysts in Figure 5 but also for Cu, Pt, Au, Ag, Pb, and Pd in the acetonitrile-based electrolyte.^18−20^

The materials’ selection studied for CO_2_RR in an acetonitrile-based solution^18−20^ only form carbonate/bicarbonate and CO if a high cathodic potential is applied. This strongly suggests that acetonitrile influences the CO_2_RR selectivity more drastically than other organic solvents. In other organic solvents, such as dimethyl formamide or dimethyl sulfoxide, the CO_2_ reduction can follow three different pathways: (i) self-coupling to form oxalate, (ii) protonation of CO_2_ to formate by residual water, or (iii) disproportionation to CO and CO_3_^2–^.^22^ The use of acetonitrile, however, seems to promote only the reaction (iii) and thus induces a “flower wire effect”, i.e., even a flower wire could be used as an electrocatalyst and would still yield the same products. The high negative potential likely leads to a break of the CH-bond. The nucleophilicity of the anion results in the formation of the carboxylated 3-aminocrotonitrile anion, which then leads to a promotion of the electroreduction of CO_2_ to dissolved CO. The reason for this is uncertain, and possible explanations could be that it proceeds either through decomposition of the carboxylated species or through activation of CO_2_ due to the presence of the carboxylated species. Thus, only a conductive electrode, such as inert GC, and a high cathodic potential are needed to reduce CO_2_ to CO in acetonitrile.

To overcome these unique properties of acetonitrile in CO_2_RR, an additional proton source, such as H_2_O or ionic liquids, could be added to the electrolyte. Recall, however, that in the case of Mo_2_C, the addition of water resulted in the oxidation of the surface and a strongly increased HER activity.^9^ Adding ionic liquids therefore seems to be the most promising avenue to harvest the intrinsic CO_2_RR characteristics of Mo_2_C.

## 4 Conclusions

In this paper, we performed spectroelectrochemical studies of CO_2_RR at Mo_2_C in an acetonitrile-based electrolyte to prevent the immediate oxidation of the surface and to suppress the HER. We show for the first time that Mo_2_C is able to reduce CO_2_ to dissolved CO. The electroreduction to CO occurs at more negative potential than carbonate/bicarbonate formation and starts simultaneously with the decomposition of acetonitrile to the 3-aminocrotonitrile anion. In the presence of CO_2_, the anion reacts instantly with CO_2_ and forms the carboxylated 3-aminocrotonitrile.

The change from an aqueous to a non-aqueous electrolyte did thus not confirm the highly praised CO_2_RR activity of Mo_2_C toward the higher reduced products. Here, the rather unique behavior of the acetonitrile-based electrolyte interferes with the theoretically predicted CO_2_RR activity of Mo_2_C. The change to ionic liquids or other organic solvents could prove the suitability of Mo_2_C as the electrocatalyst, but even then, the immediate and irreversible surface oxidation of Mo_2_C upon air exposure or through water addition would make the electrode material unsuitable for any further use.

We found, however, strong evidence that acetonitrile governs the CO_2_RR selectivity and that carbonate and dissolved CO are formed, independent of the nature of the electrocatalyst material. The acetonitrile-based electrolyte induces a “flower wire effect” on the electrode, making its catalytic selectivity almost obsolete. In other words, the electrodes merely behave as electron donors and not as catalysts that tune the selectivity to a desired product.

## Supporting Information Available

Detailed description of the experimental procedure, EC-IRRA spectra for an Ar-purged solution, s- and p-polarized light measurements, decomposition of acetonitrile in an Ar-purged electrolyte, IR transmission studies of CO in acetonitrile, calculated solvation effects of acetonitrile, vibrational frequency calculation of possible adsorbed species, and vibrational frequency calculation of the products (PDF)
