# Supplementary material for: Electroreduction of CO2 in a Non-aqueous Electrolyte—The Generic Role of Acetonitrile
Source: ACS Catal. 2023 Apr 13;13(9):5780–6. doi: 10.1021/acscatal.3c00236 (PMC10167651; doi:10.1021/acscatal.3c00236)
Supplement: Supplementary file 1 — cs3c00236_si_001.pdf [file cs3c00236_si_001.pdf]

Supplementary Information

Electro-reduction of CO_2 in non-aqueous electrolyte – the generic role of acetonitrile

Thomas Mairegger,[†1] *Haobo Li,*[2] *Christoph Grießer,*[1] *Daniel Winkler,*[1] *Jakob Filser,*[3] *Nicolas G. Hörmann,*[3] *Karsten Reuter,*[3] *Julia Kunze-Liebhäuser*[1]

[1]Department of Physical Chemistry, University of Innsbruck, Innrain 52c, 6020-Innsbruck, Austria.
[2]School of Chemical Engineering, University of Adelaide, 5005-Adelaide, Australia.
[3]Department of Theory, Fritz-Haber-Institut der Max-Planck-Gesellschaft, Faradayweg 4-6, 14195-Berlin, Germany.

*Corresponding author: Julia.Kunze@uibk.ac.at

Supplementary Note 1

Electrochemistry and electrochemical infrared spectroscopy

Cyclic voltammetry

To electrochemically investigate the oxide free electrodes in an acetonitrile (99.8 %, anhydrous, Sigma Aldrich) electrolyte, the Mo_2C electrodes were prepared[1,2] in a home-built quartz tube furnace and transported under H_2-atmosphere (> 99.999 %, Messer) into an Ar-filled glovebox (MB 200B Eco, MBraun). The cyclo voltammograms (CVs) were recorded in a commercial three electrode glass cell in a hanging meniscus configuration, utilizing an Autolab (Metrohm) potentiostat. A flame annealed carbon rod (Ultra Carbon Corporation) served as a counter electrode (CE) and a polytetrafluoroethylene (PTFE) bound activated carbon quasi reference electrode[3] was used as reference electrode (RE). Prior to each measurement, the electrolyte was purged for 20 minutes, either with Ar (> 99.999 %, Messer) or with CO_2 (5.3, 99.9993 %, Linde). The electrodes were immersed at a potential of -1.0 V versus the ferrocene/ferrocenium (99.5 %, Alfa Aesar) redox couple[4], abbreviated as $V_{Fc/Fc+}$ in the following, in an acetonitrile-based electrolyte containing 0.1 M tetrabutylammonium hexafluorophosphate ($TBAPF_6$, >98 %, Molekular). The CVs of the electrochemical CO_2 reduction reaction (CO_2RR) are shown in Figure 1a. The water content of acetonitrile, determined by Karl Fischer titration, amounts to ~ 40 ppm.

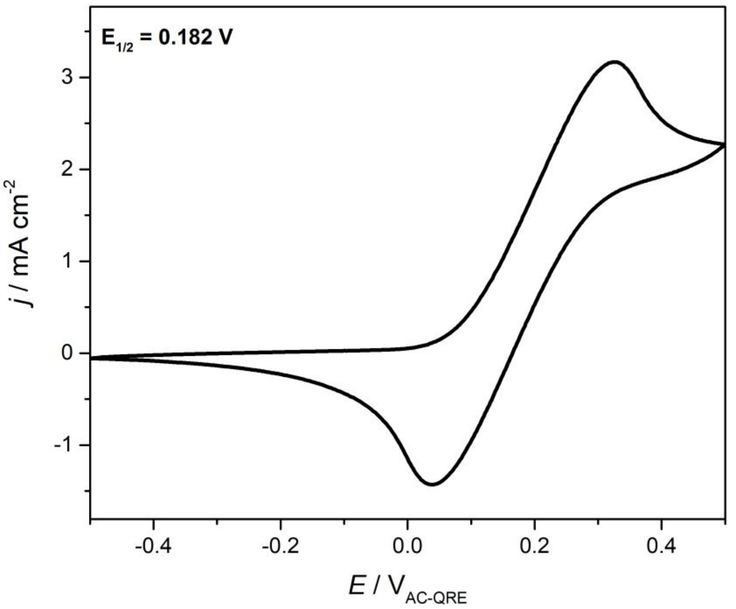

Figure S1: Cyclovoltammograms (CVs) of the ferrocene couple (ferrocene/ferrocenium (Fc/Fc+)) in acetonitrile with 0.1M $TBAPF_6$, where the half-wave potential ($E_{1/2}$) of 0.182 V versus the activated carbon quasi-reference electrode (AC-QRE) was measured in the glovebox. Scan rate = 50 mV/s.

To determine the potential of the quasi-reference electrode, the half-wave potential ($E_{1/2}$) of the ferrocene/ferrocenium couple in acetonitrile was determined as 0.182 V (Figure S1). All potentials were shifted against this value of

$$E_{Fc/Fc+} = E_{AC-QRE} + E_{1/2}$$

Electrochemical infrared reflection absorption spectroscopy (EC-IRRAS) studies

The EC-IRRAS studies were carried out using a VERTEX 70v spectrometer (Bruker) with an additional external chamber (XSA, Bruker), equipped with a liquid N_2 cooled mercury cadmium telluride (MCT) photodetector. A linear polarizer (Edmund Optics) was introduced in the optical pathway, before the IR beam entered the spectro-electrochemical cell with the use of a movable gold-coated mirror. A thin-layer cell configuration was employed to minimize the contribution of the electrolyte solution: after passing a CaF_2 hemisphere constituting the bottom of the homemade spectro-electrochemical cell, the IR beam was reflected from the surface of the WE that was pressed against the hemisphere and enters the detector with the use of a second gold-coated mirror. The cell was equipped with a carbon rod (Ultra carbon corporation) counter and the above-mentioned PTFE bound activated carbon quasi-reference electrode. To account for the strong absorption of the thin electrolyte film, the single spectra, recorded at a specific applied potential, are subtracted and normalized with the spectra recorded at a specific reference potential ($E = -1.0\ V_{Fc/Fc+}$), at which no reaction should occur, according to

$$\Delta R/R = \frac{R(E_S) - R(E_R)}{R(E_R)}$$

with $R(E_S)$ being the reflectance sample single spectra and $R(E_R)$ being the reflectance reference single spectra.

After normalization, each upward or downward facing band corresponds to consumed/disappeared or formed/accumulated species at the electrode surface, respectively. EC-IRRA spectra are taken either by alternating potential modulation, where the reference spectra are recorded between each sample spectrum, or by step potential modulation, in which the reference spectrum is taken once at the beginning of the experiment. The latter option was usually employed if not stated otherwise.

To ensure that the electrodes are oxide free, the spectro-electrochemical cell was assembled in an Ar-filled glovebox and transported under air exclusion to the IR spectrometer. Before each series of measurements, the electrolyte was purged for 20 minutes, either with Ar or CO_2,

followed by immersion of the WE at a potential of -1.0 $V_{Fc/Fc+}$ and an approach of the WE surface to the optical window.

Ar-purged electrolyte

To determine the bands originating from the electrolyte (acetonitrile with the conducting salt), measurements in deaerated electrolyte were performed (Figure S2). The bands in the wavenumber region of 3002-2880 cm^{-1} and 1500-1376 cm^{-1} are assigned to the stretching and bending modes of the conducting salt and of acetonitrile. Additionally, acetonitrile shows two strong bands at 2292 cm^{-1} and 2251 cm^{-1} and five small bands, which is in perfect agreement with the literature.[5] These bands from the electrolyte are not considered in the interpretation and discussion of the CO_2RR results determined with EC-IRRAS.

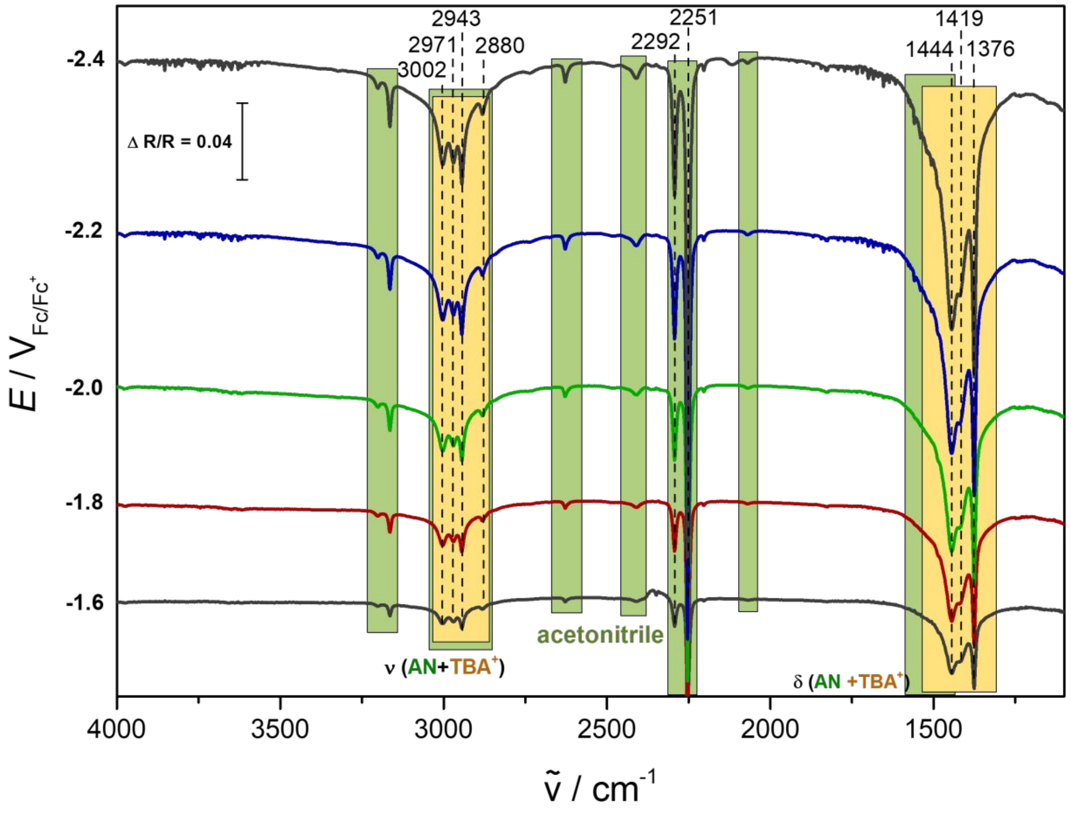

Figure S2: EC-IRRA spectra of Mo_2C recorded in Ar-purged acetonitrile with 0.1M $TBAPF_6$. The spectra show the stretching (3000-2880 cm^{-1}) and bending (1500-1376 cm^{-1}) vibrations for acetonitrile (green boxes) and the conducting salt (yellow boxes) and two distinct (2292 and 2251 cm^{-1}) as well as five smaller bands of acetonitrile (green boxes). The reference spectrum was recorded at -1.0$V_{Fc/Fc+}$.

S- and p-polarized light

Measurements with p- and s- polarized light were performed to determine if any adsorbed species is formed during the reduction reaction. The different light polarizations were implemented through manual rotation of the linear polarizer (Edmund optics) by 90°. P-polarized light probes species in solution and species adsorbed at the surface, while s-polarized light probes molecules in solution only, which is due to the destructive interference of incident and reflected beam at the electrode surface.[6]

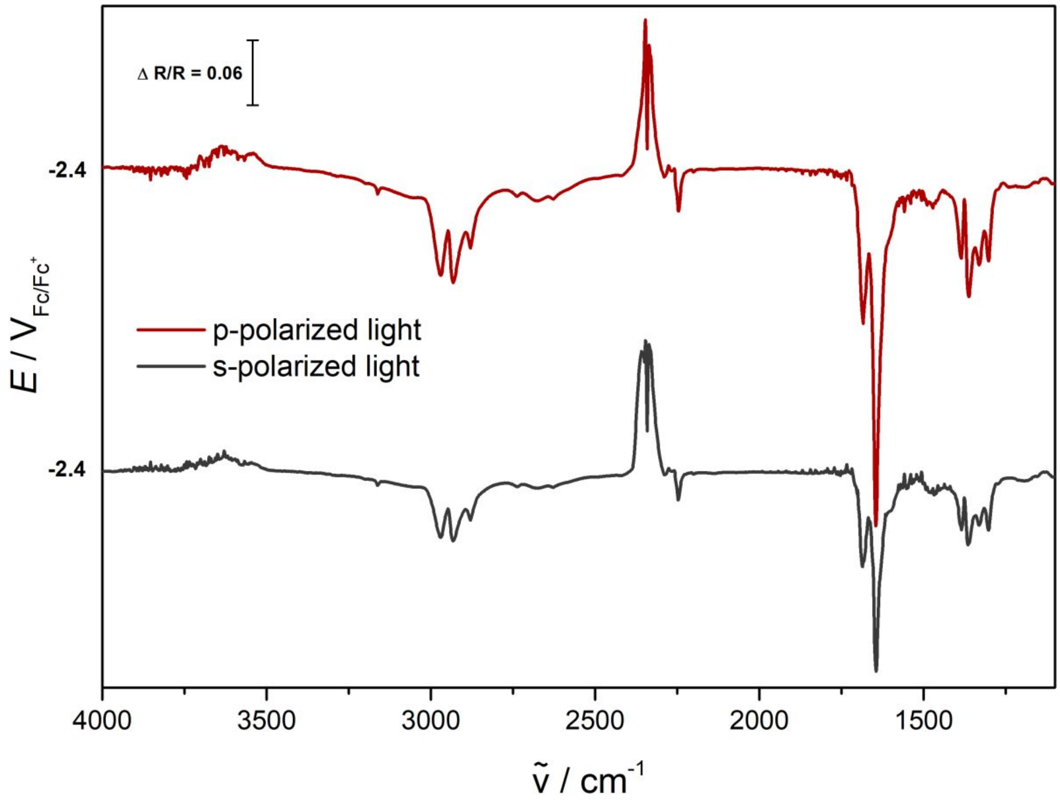

Figure S3: EC-IRRA spectra with s- and p-polarized light for the CO_2 reduction at Mo_2C in acetonitrile with 0.1M $TBAPF_6$. The reference potential was taken at -1.0$V_{Fc/Fc+}$.

EC-IRRA spectra of Mo_2C in acetonitrile with 0.1M $TBAPF_6$ at -2.4 $V_{Fc/Fc+}$ for both p- and s-light polarizations are shown in Figure S3. Both spectra show exactly the same bands, which indicates that in the case of the CO_2 reduction at Mo_2C no adsorbed species is present.

Acetonitrile decomposition

The decomposition of acetonitrile starts at a potential of -2.4 $V_{Fc/Fc+}$ with the formation of two distinct bands at 2118 cm^{-1} and 1517 cm^{-1}, along with less intense bands (Figure S4, orange boxes). The two dominant signals are associated with the formation of the 3-aminocrotonitrile anion, as known from the literature[7]. The proposed reaction route is the formation of a hydride ion, by reduction of a hydrogen atom in the metal lattice, which is reacting with acetonitrile to

its anion.[7] This acetonitrile anion nucleophilically attacks a second acetonitrile molecule and forms the 3-aminocrotonitrile anion, as shown in the inset of Figure S4.

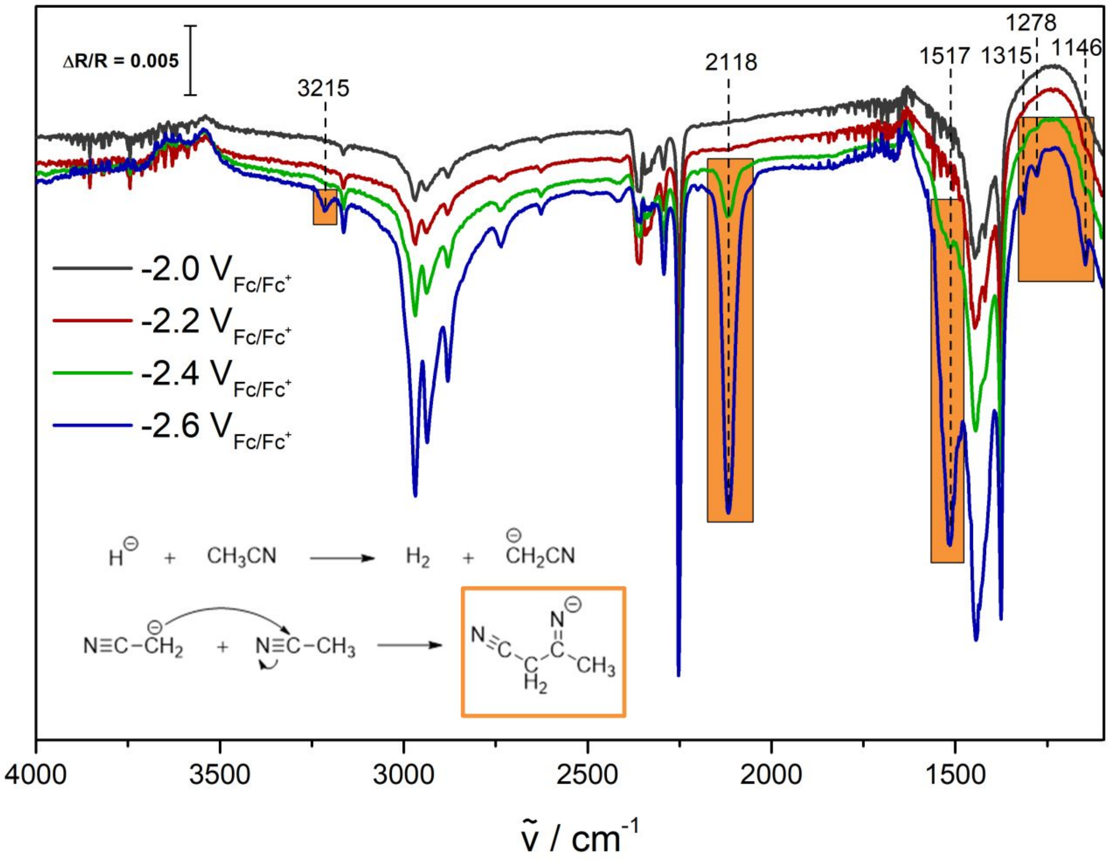

Figure S4: EC-IRRA spectra of Mo_2C in Ar-purged acetonitrile with 0.1M $TBAPF_6$. At potentials $\leq$-2.4$V_{Fc/Fc+}$, acetonitrile decomposes to the 3-aminocrotonitrile anion with its two distinct bands at 2118 cm^{-1} and 1517 cm^{-1} and less intense bands at 3215, 1315, 1278 and 1146 cm^{-1} (orange boxes). The proposed reaction pathway is depicted inside the figure (bottom left). The reference spectrum was taken at -1.0 $V_{Fc/Fc+}$.

Foley et al.[7] demonstrated that application of a more anodic potential, after the formation of the anion, leads to a protonation of the anion and formation of 3-aminocrotonitrile. In Figure S5, a potential of -2.8 $V_{Fc/Fc+}$ was applied to form the anion before increasing the potential back to its reference value of -1.0 $V_{Fc/Fc+}$. This leads to a shift of the band from 2118 cm^{-1} to 2180 cm^{-1} and to the formation of additional less intense bands at around 3400 cm^{-1} and 1600 cm^{-1} (Figure S5), which perfectly agrees with the literature.[7] Thus, we conclude that acetonitrile starts to decompose at E $\leq$ -2.4 $V_{Fc/Fc+}$ to the 3-aminocrotonitrile anion.

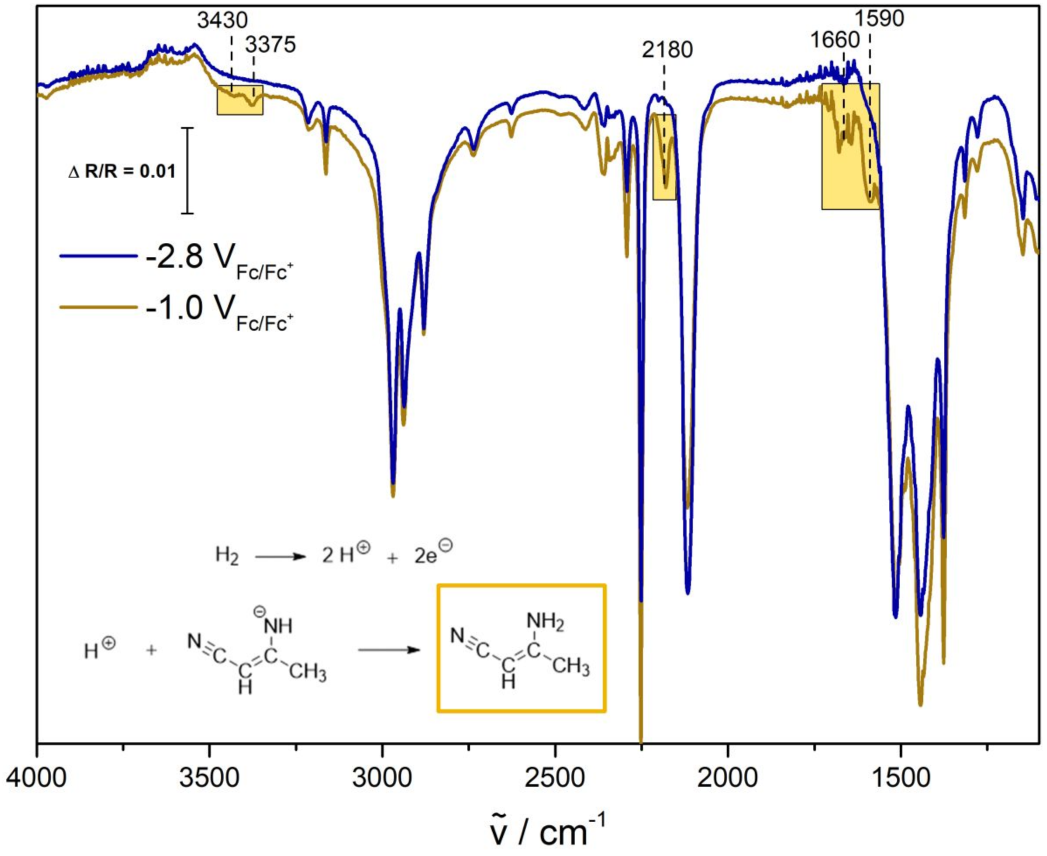

Figure S5: EC-IRRA spectra of Mo_2C in Ar-purged acetonitrile with 0.1M $TBAPF_6$. First, a potential of -2.8 $V_{Fc/Fc+}$ is applied which leads to the formation of the 3-aminocrotonitrile anion, then the potential is stepped back to the reference potential at -1.0 $V_{Fc/Fc+}$. New bands at 3430, 3375, 2180, 1660 and 1590 cm^{-1} (yellow boxes) are arising and are associated with 3-aminocrotonitrile according to the reaction pathway (bottom left). The reference spectrum was taken at -1.0 $V_{Fc/Fc+}$.

IR transmission studies

As a reference measurement, and to unambiguously determine the nature of the band at 2138 cm^{-1}, interpreted as dissolved CO, transmission cell IR studies were performed with a classical transmission cell setup (Bruker). In this setup, the electrolyte is placed between two CaF_2 plates, and the difference between CO-purged and CO-free acetonitrile IR spectra was measured. The difference spectra is shown in Figure S6 and it reveals one distinct band at 2138 cm^{-1} as reference signal for dissolved CO. The formation of dissolved CO with a corresponding band at 2138 cm^{-1} in the same acetonitrile electrolyte has been earlier shown by Figueiredo et al.[8].

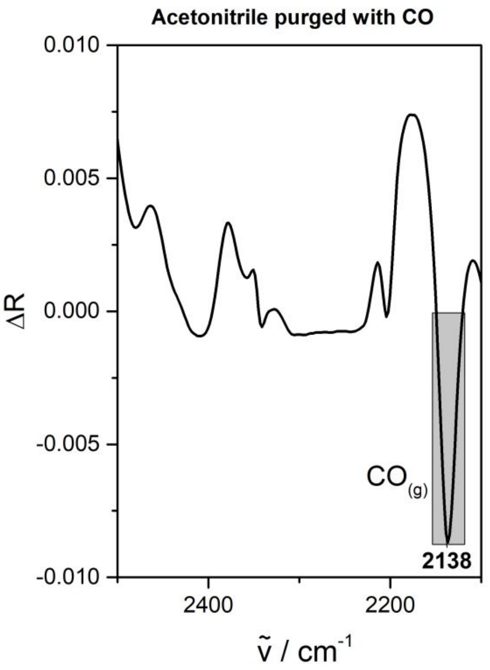

Figure S6: Transmission cell IR difference spectrum of CO-purged acetonitrile with 0.1M $TBAPF_6$. A distinct band at 2138 cm $^{-1}$, corresponding to dissolved CO is clearly visible. The difference spectrum is calculated by subtraction of the single spectra of CO with that in Ar-purged acetonitrile with 0.1M $TBAPF_6$.

Supplementary Note 2

Computational details.

Solvation effects of acetonitrile

Solvation effects of acetonitrile were considered within an implicit solvation approach using the *Environ* module[9,10] provided with QE. Parameter settings are detailed in Table S1.

Table S1: Parameter settings to the Environ module for acetonitrile.

Parameter	Value
Static permittivity (F/m)	37.5
Surface tension (dyn/cm)	47.03
Pressure (GPa)	-0.5

The adsorption free energies (G_{ad}) are calculated as:

$$G_{\mathrm{ad}} = E_{\mathrm{ad}} + ZPE + \int C_p dT - T\Delta S + \Delta G_{\mathrm{sol}}$$

where E_{ad} is the adsorption energy, $ZPE + \int C_p dT - T\Delta S$ is the vibration correction, and ΔG_{sol} is the correction of solvation effects.

The Gibbs free energies of CO_2 and H_2O molecules are calculated as:

$$\mu = E_{(\text{molecule})} + F^{\text{vib}} + k_{\text{B}}Tln(a)$$

where $E_{(\text{molecule})}$ is the total energy of an isolated molecule, $F^{\text{vib}} = ZPE + \int C_p dT - T\Delta S$ is the vibration correction, and $RTln(a)$ is the concentration correction (with k_{B} the Boltzmann constant and T the temperature). The vibration correction is calculated via DFT. The thermodynamic activity a at a low concentration is approximately equal to the molar concentration c, which for CO_2 at 100 kPa and 298 K is 279 mol·m^{-3} in acetonitrile[11], and for H_2O is 10^{-6} molar fraction (ppm level) in experimental high-purity acetonitrile.

Benchmark of vibrational frequencies on Cu(211)

Benchmark work is first done for the well-studied system Cu/acetonitrile[8]. In that work, Figueiredo et al.[8] assigned IR spectroscopy data to CO, CO_3, HCO_3 and HCO_2 surface species. Vibrational frequencies are correspondingly computed for these and a wide range of other potential C,O species (CO, CO_2, CO_3, C_2O_2, C_2O_4) and C,O,H species (COH, CHO, COOH, HCO_2, HCO_3) as summarized in Figure S7. These calculations confirm the assignments made by Figueiredo et al.[8], but exhibit an expected systematic offset in the computed absolute frequencies. Figure S8 shows a corresponding parity plot, revealing the excellent linear relation between experimental and computed data. This linear relationship extends over both data computed in vacuum and in implicit solvent, with the latter in fact leading only to a small reduction of the offset. This suggests that the offset arises predominantly from the approximate DFT functional. Henceforth the linear relationship obtained here is employed to correct for this systematic error. Table S2 compiles the thus corrected absolute frequencies and demonstrates that the remaining error in absolute frequencies after this correction is on the order of a few percent.

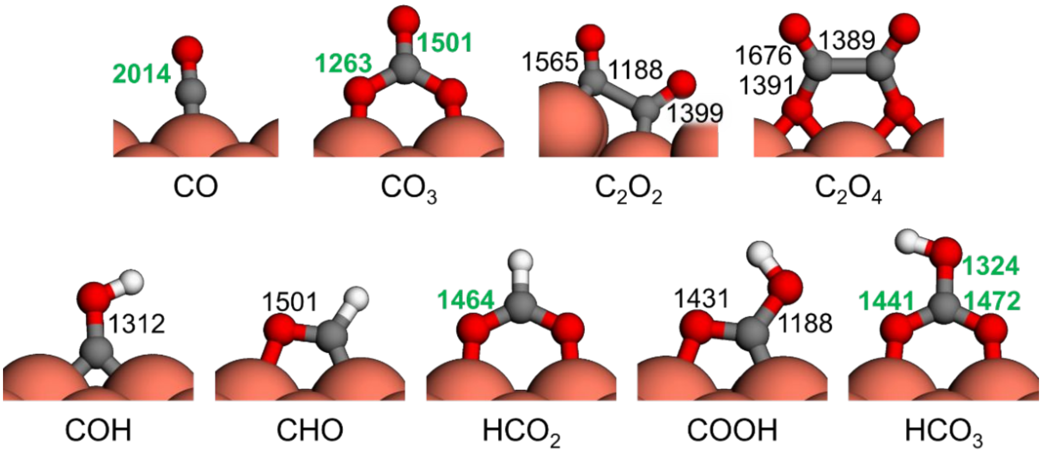

Figure S7**:** Adsorption configurations of various C-containing species on Cu(211) in acetonitrile. DFT-calculated vibrational frequencies in acetonitrile are marked at each C-O or C-C bond. Green values indicate the surface species in the assignment made by Figueiredo and coworkers[8]. Large orange atoms: Cu; grey atoms: C; red atoms: O; white atoms: H.

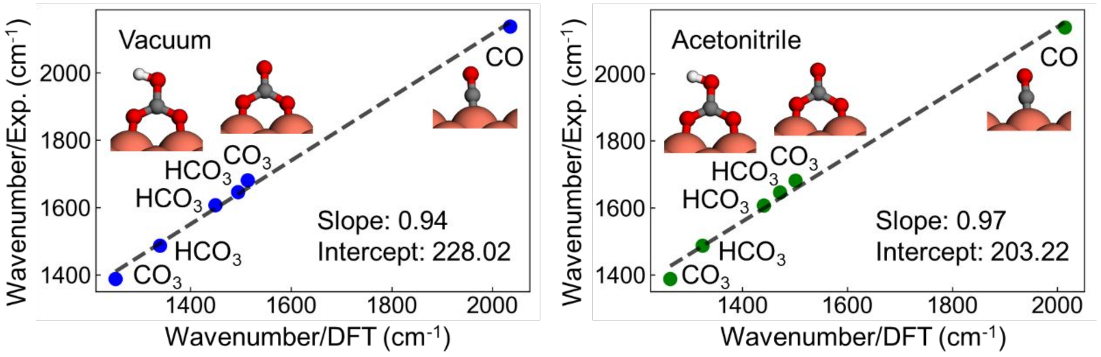

Figure S8: Benchmark of experimental vibrational frequencies of C-containing species on Cu(211) with theoretical frequencies calculated in vacuum (left) and in acetonitrile (right). The fitted linear relationship between experimental and computational data is shown as black dashed line, with the fitting parameters given as inset.

Table S2: Assignment of the IR frequencies for C-O stretching of CO_2 reduction species on Cu electrodes in acetonitrile. Computed values include the offset correction.

Wave number (Exp.) (cm^{-1})	Species (Ref)[8]	Wave number (Theory) (cm^{-1})	Species (this work)	Error (%)
2138	**CO**	2153	**CO**	0.70
1681	**CO_3**	1657	**CO_3**	-1.43
1646	**CO_3**	1628	**HCO_3/HCO_2**	-1.09
1607	**HCO_3**	1598	**HCO_3**	-0.56
1487	**HCO_2**	1485	**HCO_3**	-0.13
1452	**HCO_2**	1473	**COH**	1.45
1388	**HCO_2**	1426	**CO_3**	2.74

Adsorption of CO_2 reduction species on $Mo_2C(110)$

The same C-containing adsorbates as in the benchmark are calculated on the C-rich $Mo_2C(110)$ surface in an acetonitrile environment. A variety of adsorption sites and configurations is considered to find the most stable adsorption mode (in the employed sign convention here reflected by a most negative G_{ad}). The corresponding results are summarized in Table S3.

Table S3: Adsorption sites and configurations, adsorption free energy (G_{ad}), vibrational frequencies of C-O (and C-C for C_2O_2, C_2O_4) stretching modes (υ), and solvation correction in acetonitrile (ΔG_{sol}) of various C-containing surface species on C-rich $Mo_2C(110)$. Tentative assignments to experimental fingerprints (blue) are marked in green (see text). Data for configurations corresponding to the most stable adsorption mode are in bold.

**CO**				
**Site**	**G_{ad} (eV)**	**uC-O (cm^{-1})**	**ΔG_{sol} (eV)**	**Top view**
**C-top**	**-0.91**	**2144**	**-0.37**	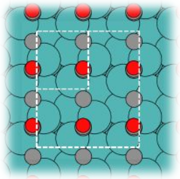
C-top	0.48	2139	-0.39	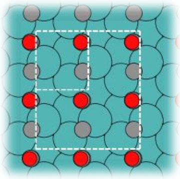
Mo-top	-0.35	2158	-0.36	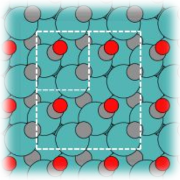
**CO_2**				
**C-top**	**-0.56**	**1807**<br>**1494**	**-0.08**	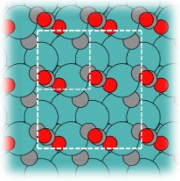
C-top	1.16	1828<br>1507	0.03	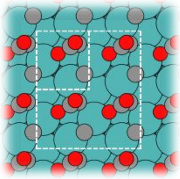

Mo-bridge	0.82	1800	0.08	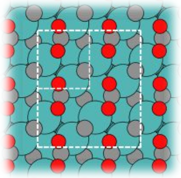
		1752		
CO_3				
MoC-bridge	1.60	1964	0.45	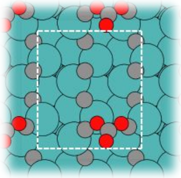
		1626		
		1622		
Mo-bridge	-0.92	1872	0.29	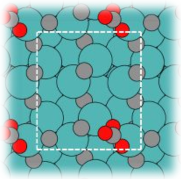
		1618		
		1592		
**Mo-bridge**	**-1.10**	**1860**	**0.31**	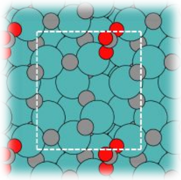
		**1554**		
		**1553**		
**Mo-hollow**	**-1.17**	**1658**	**0.48**	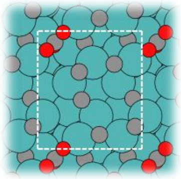
		**1630**		
		**1515**		
C_2O_2				
**MoC-bridge**	**-0.23**	**1750**	**-0.84**	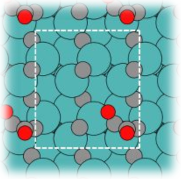
		**1528**		
		**1482** (C-C)		
MoC-bridge	0.19	1439	-0.83	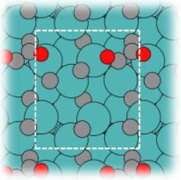
		1409		
		1240 (C-C)		
**MoC-bridge**	**-0.19**	**1697**	**-0.87**	
		**1382**		

		**1460 (C-C)**		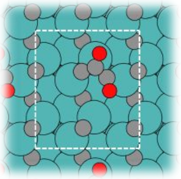
C-bridge	-0.14	1780<br>1353<br>1465 (C-C)	-0.96	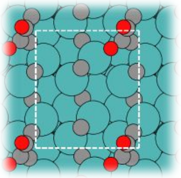
**C_2O_4**				
MoC-bridge	-0.02	1911<br>1806<br>1580<br>1517<br>1594 (C-C)	0.05	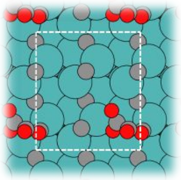
MoC-bridge	1.38	1910<br>1636<br>1572<br>1567<br>1584 (C-C)	0.09	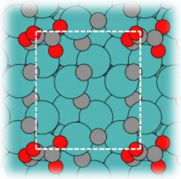
Mo-bridge	-0.39	1937<br>1889<br>1588<br>1580<br>1593 (C-C)	-0.10	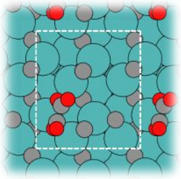
**Mo-bridge**	**-0.64**	**1838**<br>**1825**<br>**1544**<br>**1527**<br>**1538** (C-C)	**-0.19**	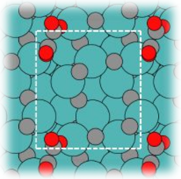
**HCO_3**				
Mo-bridge	-0.82	1613<br>1561<br>1490	0.33	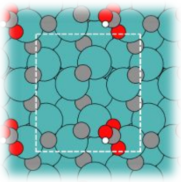
**Mo-bridge**	**-1.29**	**1563**<br>**1544**<br>**1460**	**0.38**	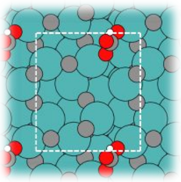

Mo-hollow	-0.17	1720<br>1585<br>1531	0.28	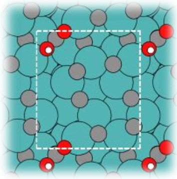
**HCO_2**				
Mo-bridge	-0.98	1708<br>1525	0.06	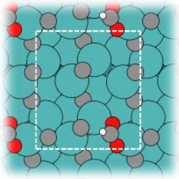
**Mo-bridge**	**-1.53**	**1578**<br>**1575**	**0.07**	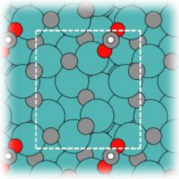
**COOH**				
**MoC-bridge**	**-0.92**	**1559**<br>**1450**	**-0.03**	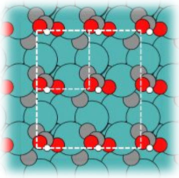
MoC-bridge	0.54	1577<br>1448	-0.05	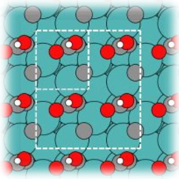
Mo-bridge	0.27	1536<br>1361	0.10	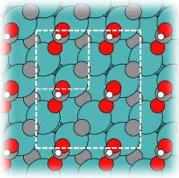
**CHO**				
**MoC-bridge**	**-1.08**	**1520**	**-0.36**	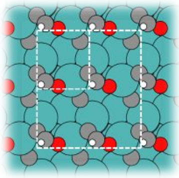

MoC-bridge	0.17	1474	-0.27	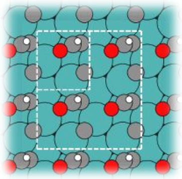
Mo-bridge	0.33	1599	-0.23	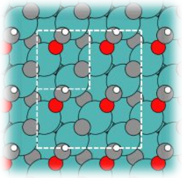
**COH**				
MoC-bridge	-0.64	1562	-0.29	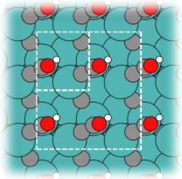
**MoC-bridge**	**-1.19**	**1589**	**-0.40**	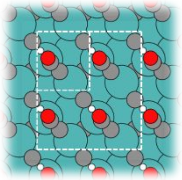
C-bridge	-0.99	1358	-0.34	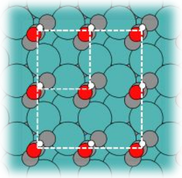

For almost all adsorbates there is one energetically preferred adsorption configuration. The only exceptions are C_2O_2 and CO_3, where the second most stable configuration is only by 40 meV and 70 meV weaker bound, respectively. We consider the latter small energetic difference within the uncertainty of our approach and correspondingly also keep this second most stable configuration in mind for the assignment to the experimental fingerprints.

Vibrational frequency calculation of the products

The vibrational frequencies of the products (3-aminocrotonitrile anion, 3-aminocrotonitrile, carboxylated acetonitrile and carboxylated 3-aminocrotonitrile anion) were calculated in each supercell with a side length of 20 Å (Table S4). The calculated frequencies (ν_{DFT}) are offset corrected according to following equations

$$\nu_{Theo} = s \cdot \nu_{DFT} \tag{S1}$$

$$s = \nu_{Exp}\,(I) / \nu_{DFT}\,(I) \tag{S2}$$

where ν_{DFT} is the calculated vibrational frequency, ν_{Theo} the offset corrected vibrational frequency and *s* the factor for the offset correction.

Table S4: Vibrational frequency calculation of (I) 3-aminocrotonitrile anion, (II) 3-aminocrotonitrile, (III) carboxylated acetonitrile and (IV) carboxylated 3-aminocrotonitrile anion in acetonitrile. The frequencies are offset corrected according to equation S1 and S2.

**Molecule**	ν_{DFT} / cm^{-1}	ν_{Exp} / cm^{-1}	ν_{Theo} / cm^{-1}
**I**	2152.8	2120	**2120.0**
**II**	2210.0		**2176.3**
**III**	2269.8		2235.2
**IV**	2188.0		**2154.7**

References

1. Griesser, C., Li, H., Wernig, E.-M., Winkler, D., Shakibi Nia, N., Mairegger, T., Götsch, T., Schachinger, T., Steiger-Thirsfeld, A., Penner, S., Wielend, D., Egger, D., Scheurer, C., Reuter, K. & Kunze-Liebhäuser, J. True Nature of the Transition-Metal Carbide/Liquid Interface Determines Its Reactivity. *ACS Catal.* **11,** 4920–4928; 10.1021/acscatal.1c00415 (2021).
2. Winkler, D., Dietrich, V., Griesser, C., Nia, N. S., Wernig, E.-M., Tollinger, M. & Kunze-Liebhäuser, J. Formic acid reduction and CO_2 activation at Mo_2C: The important role of surface oxide. *Electrochem Sci Adv.* **2**, e2100130; 10.1002/elsa.202100130 (2022).
3. Auer, A. & Kunze-Liebhäuser, J. A universal quasi-reference electrode for in situ EC-STM. *Electrochem. commun.* **98,** 15–18; 10.1016/j.elecom.2018.11.015 (2019).
4. Gritzner, G. & Kuta, J. Recommendations on reporting electrode potentials in nonaqueous solvents (Recommendations 1983). *Pure Appl. Chem.* **56,** 461–466; 10.1351/pac198456040461 (1984).
5. National Institute of Standards and Technology. Acetonitrile. Available at https://webbook.nist.gov/cgi/cbook.cgi?ID=75-05-8&Type=IR-SPEC&Index=QUANT-IR,3#IR-SPEC (2022).
6. Zamlynny, V. & Lipkowski, J. Quantitative SNIFTIRS and PM IRRAS of Organic Molecules at Electrode Surfaces. In *Advances in Electrochemical Science and Engineering,* edited by H. Gerischer & C. W. Tobias (Wiley1995), Vol. 9, pp. 315–376.
7. Foley, J. K., Korzeniewski, C. & Pons, S. Anodic and cathodic reactions in acetonitrile/tetra-n-butylammonium tetrafluoroborate: an electrochemical and infrared spectroelectrochemical study. *Can. J. Chem.* **66,** 201–206; 10.1139/v88-033 (1988).
8. Figueiredo, M. C., Ledezma-Yanez, I. & Koper, M. T. M. In Situ Spectroscopic Study of CO_2 Electroreduction at Copper Electrodes in Acetonitrile. *ACS Catal.* **6,** 2382–2392; 10.1021/acscatal.5b02543 (2016).
9. Andreussi, O., Dabo, I. & Marzari, N. Revised self-consistent continuum solvation in electronic-structure calculations. *Chem. Phys.* **136,** 64102; 10.1063/1.3676407 (2012).
10. Andreussi, O., Hörmann, N. G., Nattino, F., Fisicaro, G., Goedecker, S. & Marzari, N. Solvent-Aware Interfaces in Continuum Solvation. *J. Chem. Theory Comput.* **15,** 1996–2009; 10.1021/acs.jctc.8b01174 (2019).
11. Gennaro, A., Isse, A. A. & Vianello, E. Solubility and electrochemical determination of CO_2 in some dipolar aprotic solvents. *J. electroanal. chem.* **289,** 203–215; 10.1016/0022-0728(90)87217-8 (1990).
